# Abnormal Ergosterol Biosynthesis Activates Transcriptional Responses to Antifungal Azoles

**DOI:** 10.3389/fmicb.2018.00009

**Published:** 2018-01-17

**Authors:** Chengcheng Hu, Mi Zhou, Wenzhao Wang, Xianyun Sun, Oded Yarden, Shaojie Li

**Affiliations:** ^1^State Key Laboratory of Mycology, Institute of Microbiology, Chinese Academy of Sciences, Beijing, China; ^2^College of Life Sciences, University of Chinese Academy of Sciences, Beijing, China; ^3^Department of Plant Pathology and Microbiology, The Robert H. Smith Faculty of Agriculture, Food and Environment, The Hebrew University of Jerusalem, Rehovot, Israel

**Keywords:** azoles, stress response, sterol 14α-demethylase, C-8 sterol isomerase, efflux pump, sterol intermediate, *tcu-1* promoter

## Abstract

Fungi transcriptionally upregulate expression of azole efflux pumps and ergosterol biosynthesis pathway genes when exposed to antifungal agents that target ergosterol biosynthesis. To date, these transcriptional responses have been shown to be dependent on the presence of the azoles and/or depletion of ergosterol. Using an inducible promoter to regulate *Neurospora crassa erg11*, which encodes the major azole target, sterol 14α-demethylase, we were able to demonstrate that the CDR4 azole efflux pump can be transcriptionally activated by ergosterol biosynthesis inhibition even in the absence of azoles. By analyzing ergosterol deficient mutants, we demonstrate that the transcriptional responses by *cdr4* and, unexpectedly, genes encoding ergosterol biosynthesis enzymes (*erg* genes) that are responsive to azoles, are not dependent on ergosterol depletion. Nonetheless, deletion of *erg2*, which encodes C-8 sterol isomerase, also induced expression of *cdr4*. Deletion of *erg2* also induced the expression of *erg24*, the gene encoding C-14 sterol reductase, but not other tested *erg* genes which were responsive to *erg11* inactivation. This indicates that inhibition of specific steps of ergosterol biosynthesis can result in different transcriptional responses, which is further supported by our results obtained using different ergosterol biosynthesis inhibitors. Together with the sterol profiles, these results suggest that the transcriptional responses by *cdr4* and *erg* genes are associated with accumulation of specific sterol intermediate(s). This was further supported by the fact that when the *erg2* mutant was treated with ketoconazole, upstream inhibition overrode the effects by downstream inhibition on ergosterol biosynthesis pathway. Even though *cdr4* expression is associated with the accumulation of sterol intermediates, intra- and extracellular sterol analysis by HPLC-MS indicated that the transcriptional induction of *cdr4* did not result in efflux of the accumulated intermediate(s). This study demonstrates, by detailed genetic and chemical analysis, that transcriptional responses by a major efflux pump and genes of the ergosterol biosynthesis pathway to ergosterol biosynthesis inhibitors can be independent of the presence of the drugs and are linked with the accumulation of ergosterol intermediate(s).

## Introduction

Fungal diseases in crops significantly contribute to yield loss and mycotoxin contaminations (Dean et al., 2012), while invasive fungal infections in immunodeficient patients are often the cause for mortality (Brown et al., 2012). For decades, antifungal azoles have been prominently used in the control of detrimental fungi in the clinic and in agriculture due to their broad antifungal spectra, low toxicity and low cost. However, azole resistance, which accompanies the long-term drug use of these compounds, has made fungal pathogen control more challenging in recent years in both clinic and agriculture (Cowen et al., 2014; Price et al., 2015).

Antifungal azoles block ergosterol biosynthesis by inhibiting sterol 14α-demethylase. Inhibition by azoles commonly leads to depletion of ergosterol and accumulation of other sterols, such as lanosterol, eburicol and the toxic 14α-methyl-3,6-diol, within fungal cell membranes (Watson et al., 1989; Shapiro et al., 2011; Chen et al., 2016). Two important systems shown to be critical to azole resistance are the efflux pump and ergosterol homeostasis systems. The efflux pump system, which is comprised of pumps located in the cell membrane and their regulators, is present in almost all species and plays an important roles in drug resistance in bacteria, fungi and human cancer cells by efflux of the drugs (Golin et al., 2007; Blair et al., 2014; Cowen et al., 2014; Paul and Moye-Rowley, 2014; Tanwar et al., 2014; Kathawala et al., 2015). Ergosterol homeostasis, which is tightly maintained by several important regulators, is imperative for ergosterol biosynthesis and cell membrane functions (Bien and Espenshade, 2010; Maguire et al., 2014). Upon azole stress, fungi can respond with a change in the transcription of a variety of genes. The most commonly observed azole-responsive genes include the gene encoding the azole target sterol 14α-demethylase and those encoding efflux pumps such as the *Saccharomyces cerevisiae* Pdr5p, the *Candida albicans* Cdr1/2p and Mdr1p and the *N. crassa* CDR4 (NCU05591), as well as other genes in ergosterol biosynthesis, including *ERG2* (encoding C-8 sterol isomerase), *ERG5* (encoding C-22 sterol desaturase), *ERG6* (encoding C-24 sterol methyl transferase) and *ERG24* (encoding C-14 sterol reductase) in *S. cerevisiae* and their homologs in other fungi (Agarwal et al., 2003; Liu et al., 2005, 2010; Ferreira et al., 2006; Yu et al., 2007; Hoehamer et al., 2010; Florio et al., 2011; Sun et al., 2014), indicating the two systems are transcriptionally activated by azoles. In clinical and agricultural azole-resistant isolates, overexpression of sterol 14α-demethylase or azole efflux pumps are among the major causes for azole resistance (White, 1997; Perea et al., 2001; Perea et al., 2002; Redding et al., 2003; Cools et al., 2013; Price et al., 2015). The responsive C-22 sterol desaturase coding gene was also demonstrated to be important for the basal resistance to azoles in *N. crassa* and *Fusarium verticillioides* (Sun et al., 2013). In addition, transcription factors, such as Tac1p that regulates the azole efflux pumps and Upc2p that regulates ergosterol biosynthesis genes in *C. albicans*, govern the expression of many azole-responsive genes and confer azole resistance (Coste et al., 2004; Coste et al., 2006; Dunkel et al., 2008; Heilmann et al., 2010; Flowers et al., 2012; Vasicek et al., 2014). The phenomena described above indicate that activation of the transcriptional responses to antifungal azoles is an important strategy for azole resistance.

For transcriptional responses of azole efflux pumps, the azole drug itself was shown to activate gene expression of azole efflux pumps. In *S. cerevisiae*, the transcription factors Pdr1p and Pdr3p, which regulate expression of efflux pumps, can be activated by directly interacting with xenobiotics, including ketoconazole, and thus might act as sensors of xenobiotics (Thakur et al., 2008). However, it is unknown whether the sterol content changes caused by azole inhibition can result in the activation of azole efflux pumps.

For azole-activated transcriptional responses by genes for ergosterol biosynthesis, several lines of evidence suggest that ergosterol depletion might be a cause. In *Schizosaccharomyces pombe*, it was reported that ergosterol depletion activated the expression of ergosterol biosynthesis genes and their regulator Sre1p (Hughes et al., 2005; Porter et al., 2010). In *S. cerevisiae*, transcription factor Upc2p regulates expression of ergosterol biosynthesis genes. Upc2p directly interacts with ergosterol and its activity was regulated by ergosterol levels (Yang et al., 2015). Thus, azole-caused ergosterol depletion might activate transcriptional response via Upc2p. Other conditions causing ergosterol depletion, by ergosterol biosynthesis inhibitors other than azoles, or mutation of some genes in ergosterol biosynthesis pathway, also activate the expression of genes for ergosterol biosynthesis in *S. cerevisiae* and *Candida* species (Bammert and Fostel, 2000; Henry et al., 2000). All above lines of evidence suggest that ergosterol depletion resulted from azole stress might be a cause for the transcriptional responses by ergosterol biosynthesis genes, but some direct experimental data in other fungi are still required for making a definite conclusion. In addition, the accumulated toxic 14α-methyl-3,6-diol was also thought to cause membrane stresses and activate stress responses with unclear mechanisms (Watson et al., 1989; Shapiro et al., 2011). Thus, the accumulation of sterol intermediates is also possible to induce the transcriptional responses.

In this study, based on genetic and chemical analysis in *N. crassa*, we demonstrate that abnormal ergosterol biosynthesis, causing accumulation of sterol intermediates but not ergosterol depletion, can result in transcriptional responses by efflux pump CDR4, as well as ergosterol biosynthesis genes, similar to those under azole stress.

## Materials and Methods

### Strains and Culture Conditions

**FIGURE 1:**
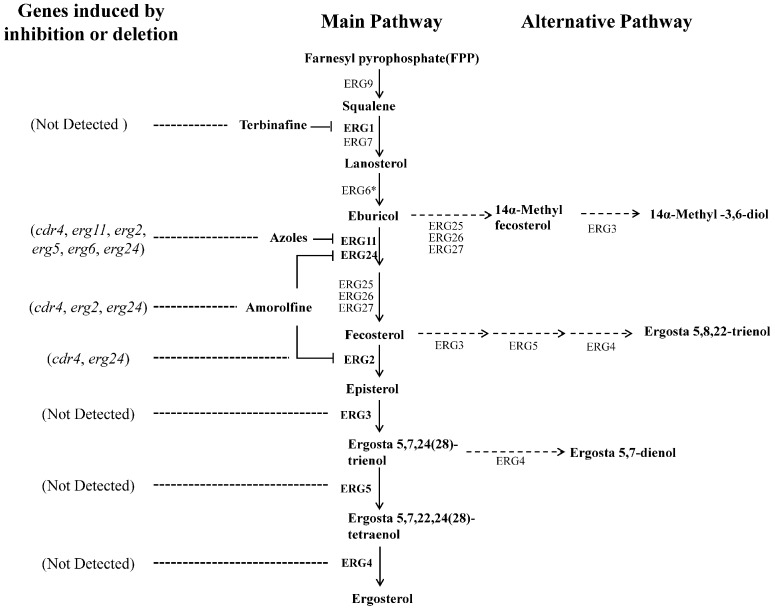
Suggested ergosterol biosynthesis pathway in *N. crassa* and transcriptional responses to disruption at certain steps. Each enzyme relates to one step of the ergosterol biosynthesis pathway in *N. crassa*. The most marked difference between the *N. crassa* and *S. cerevisiae* is the step catalyzed by ERG6 (marked by an asterisk). This step follows the reactions catalyzed by ERG25, ERG26 and ERG27 in yeast. Corresponding genes encoding enzymes in bold were analyzed in this study. Dotted arrows show the alternative biosynthesis pathways when the main pathway is impaired. Dotted lines showed the genes transcriptional induced by ergosterol biosynthesis inhibitors or *erg* gene deletion.

*Neurospora crassa* strains used in this study and their sources are listed in **Table 1**. Designations of ergosterol biosynthesis genes followed a previous study in *N. crassa*, in which all of the genes were named with those of their orthologs in yeast (Sun et al., 2013). A suggested partial ergosterol biosynthesis pathway in *N. crassa* is shown in **Figure 1**. However, as different *N. crassa* designations have been used by various authors in the past, we have compiled a list of comparative *erg* gene designations for clarification (Supplementary Table 1). Strains were cultured on Vogel’s medium with 1.5% agar [Vogel’s minimum medium, supplemented with 2% (w/vol) sucrose for slants or glucose for plates] (Vogel, 1956). Liquid Vogel’s minimum medium supplemented with 2% glucose was used for preparing samples for RNA, protein and sterol extraction. Sorbose plates (Vogel’s minimum medium, supplemented with 20 g/L sorbose, 0.5 g/L fructose and 0.5 g/L glucose) were used for electroporation transformation (Davis and de Serres, 1970). All cultures were grown at 28°C.

**Table 1:** Strains used in this study.

Strain	Genotype (Locus No., Protein encoded)	Resource
*N. crassa* FGSC #4200	74-ORs-6a	FGSC
*N. crassa bd ku70^RIP^*	Ku70^*RIP*^; Ras-1^*bd*^	He et al., 2006
*N. crassa* P*tcu-1*::*erg11*	P*tcu-1*::5 × myc-6 × his::*erg11*; Ku70^*RIP*^; Ras-1^*bd*^	This study
*N. crassa* P*erg11*::*erg11*	P*erg11*::5 × myc-6 × his::*erg11*; Ku70^*RIP*^; Ras-1^*bd*^	This study
*N. crassa* Δ*erg2*	Δ*erg2* (NCU04156, C-8 sterol isomerase)	This study
*N. crassa* Δ*erg3*	Δ*erg3* (NCU06207, C-5 sterol desaturase)	FGSC
*N. crassa* Δ*erg4*	Δ*erg4* (NCU01333, C-24(28) sterol reductase)	FGSC
*N. crassa Δerg5*	Δ*erg5* (NCU05278, C-22 sterol desaturase)	FGSC
*N. crassa Δcdr4*	Δ*cdr4* (NCU05591, ABC transporter CDR4)	FGSC


Antifungal compounds were added as needed. The antifungal agents include ketoconazole (KTC, Sigma), amorolfine (AMOR, Tokyo Chemical Industry) and terbinafine (TERB, J&K), dissolved in DMSO, Methanol and Methanol, respectively. Media were amended with the drugs to a final concentration of 2 mg/L, 0.375 mg/L and 3 mg/L, respectively, which resulted in *N. crassa* growth inhibition of at least 50%. Hygromycin B (Amresco) at a final concentration of 150 mg/L was used for screening fungal transformants and added as required.

### Mutant Construction

**FIGURE 2:**
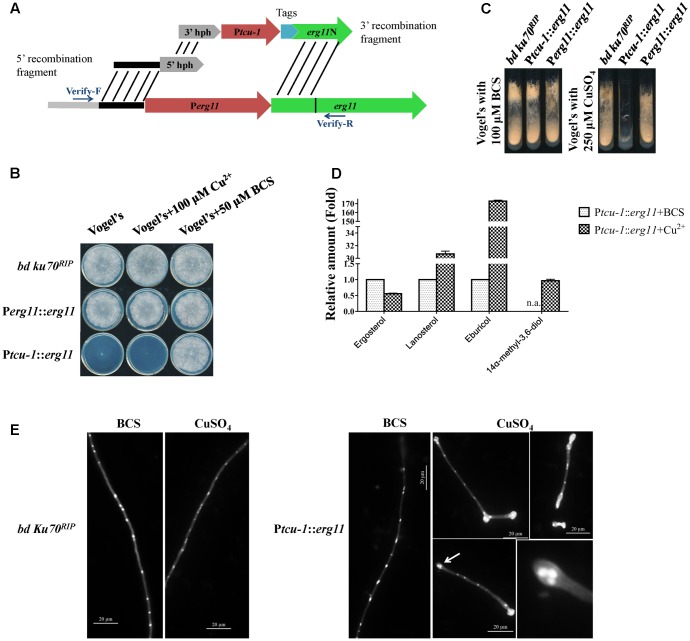
*erg11* is an essential gene in *N. crassa*. **(A)** Schematic diagram of the process of P*tcu-1*::*erg11* (NCU02624, encoding sterol 14α-demethylase) strain construction. **(B,C)** *erg11* is required for fungal growth. Conidia of *N. crassa bd Ku70^RIP^* strain (the parental strain), P*tcu-1*::*erg11* mutant and the complemented P*erg11*::*erg11* mutant were inoculated on Vogel’s plates **(B)** and Vogel’s slants **(C)** supplemented with or without CuSO_4_ or Cu^2+^ chelator BCS and grown at 28°C for 7 days **(B)** or 30 h **(C)**, respectively. **(D)** *erg11* inactivation resulted in sterol content changes in *N. crassa*. After grown for 13.5 h in liquid Vogel’s medium amended with BCS, the mycelium was then transferred to liquid Vogel’s medium amended with BCS and CuSO_4_ and treated for 22 h. The sterols were then extracted and subjected to HPLC-MS analysis. The abundance of sterols was calculated on the basis of the chromatogram peak area and normalized using an internal control and sample weight. The results presented here are means of two biological replicates. **(E)** Conidia of *bd Ku70^RIP^* and P*tcu-1*::*erg11* were germinated in Vogel’s minimal medium supplemented with BCS or CuSO_4_ for 10 h. Hyphae were imaged after fixing and staining with calcofluor white and DAPI. Clustered nuclei are indicated by arrows and enlarged (below panel on the right).

In order to generate a strain in which transcription of *erg11* (NCU02624, encoding sterol 14α-demethylase, **Figure 1**) could be controlled, the native promoter of *erg11* was replaced with the copper responsive promoter P*tcu-1*, based on a previously described strategy (Lamb et al., 2013). The construct diagram was shown in **Figure 2A**. First, a dominant selectable marker cassette was fused to the 5′ terminus of the *tcu-1* promoter. The appropriate fragments of the *tcu-1* promoter (1716 bp) and the Hygromycin B resistance gene *hph*, flanked by the *trpC* promoter and terminator (2169 bp), were amplified using primers Ptcu-1F/R and HphR-F/R, respectively (Supplementary Table 2). A homologous recombination technology-based kit (ClonExpress MultiS One Step Cloning Kit; Vazyme, Nanjing, China) was used to insert the two fragments into pBluescript II SK, resulting in the formation of plasmid pSKII-hph-Ptcu-1. Second, a split marker method was used to construct the mutant and two rounds of PCR were performed to obtain the recombination fragments. In the first round, four fragments, comprised of the 565 bp upstream homologous region of *erg11*, 834 bp downstream homologous region of *erg11* (*erg11*N), upstream half of hph-Ptcu-1 and downstream half of hph-Ptcu-1, were amplified using primer pairs exPerg-up-F/R, exPerg-down-F/R, hph-Ptcu-up-F/R and hph-Ptcu-down-F/R, respectively (Supplementary Table 2). In addition, the downstream homologous region of *erg11* was amplified from a construct containing a 5 × myc-6 × his-tagged version of the gene (Hu et al., unpublished). In the second round of amplification, the two upstream fragments and the two downstream fragments were joined, by fusion PCR, using primer pairs exPerg-up-F, hph-Ptcu-up-R and hph-Ptcu-down-F, exPerg-down-R, respectively (Supplementary Table 2) and resulted in 5′ recombination fragment and 3′ recombination fragment (**Figure 2A**). The obtained two recombination fragments were purified and then directly co-transformed into an available strain that was Ku70 deficient in a Ras-1*^bd^* background by electroporation (He et al., 2006). Transformants were screened on plates containing 50 mg/L bathocuproinedisulfonic acid (BCS, Sigma) and 150 mg/L Hygromycin B and verified by PCR using primer pair exPerg-vF/R (Supplementary Table 2). After grown for several generations on slants containing 150 mg/L Hygromycin B, homozygous strains were screened and verified by PCR and used for the following experiments.

The *erg11* inactivation strain was complemented by replacing the *tcu-1* promoter with the native promoter of *erg11*. Briefly, three fragments, comprised of the terminator region of *trpC*, native promoter of *erg11* and 5 × myc-6 × his tagged coding region of *erg11*, were amplified using primers TtrpC-F/R, Perg11F/R, and exPerg-down-F’/R’, respectively (Supplementary Table 2). The three fragments were then joined, by fusion PCR, using primer pairs TtrpC-F and exPerg-down-R’ (Supplementary Table 2). The obtained fragment was purified and then directly transformed into the *erg11* inactivation strain P*tcu-1*::*erg11*. Since the P*tcu-1*::*erg11* strain cannot grow on media containing Cu^2++^, Cu^2+^ were used to screen the transformants. The genetic alterations introduced to the transformants were then verified by PCR.

The *erg2* gene (NCU04156, encoding C-8 sterol isomerase, **Figure 1**) was deleted as described (Colot et al., 2006). Briefly, a 1153 bp promoter region and 1304 bp terminator region of *erg2*, as well as a 1416 bp fragment containing the Hygromycin B resistant gene, were amplified using primer pairs Perg2-up-F/R, Perg2-down-F/R and hph-F/R, respectively (Supplementary Table 2). The three fragments were subjected to fusion PCR using primers Perg2-up-F and Perg2-down-R (Supplementary Table 2). The obtained fragment was purified and then transformed into the wild-type strain FGSC #4200 by electroporation transformation as previously reported (Wang et al., 2015). Transformants were screened on plates containing 150 mg/L Hygromycin B and the gene replacement was verified by PCR.

### Phenotypic Analysis of Mutants

For macroscopic and microscopic analysis of mutants, Vogel’s minimal medium dishes were inoculated with 2 μL aliquot of conidial suspension (2 × 10^6^ /mL) and the strains were cultured at 28°C. Growth media either contained or lacked CuSO_4_ or was amended with BCS supplements. The final concentrations of CuSO_4_ and BCS were 100 and 50 μM, respectively. Growth was documented after growing for at least 28 h and colony edges were subjected to microscopic examination. Spore suspensions were also inoculated onto Vogel’s slants with or without CuSO_4_ or BCS supplements and incubated at 28°C. After 5–7 days, aerial hyphal formation and conidiation were analyzed.

Calcofluor white (0.5 g/L, Fluka, Sigma) and DAPI (Sango Biotech) were used to visualize cell wall and nucleus, respectively. Briefly, conidia of each strain were inoculated into liquid media to a final concentration of 2 × 10^6^ conidia per mL and allowed to germinate and grow for 10 h. The cultures were centrifuged and the pellets were fixed in 10% formalin solution for 30 min. The fixed mycelia were then washed with phosphate-buffered saline (PBS) for 2–3 times. After that, 5 μL of concentrated culture was mixed with calcofluor white and DAPI to a final concentration of 500 μg/L and 3 μg/mL, respectively, and stained for 10 min. Fluorescent microscopic observation was performed with a Zeiss ImagerA2 microscope equipped with a digital camera and DAPI Zeiss filter set was used for visualizing.

### RNA Extraction and Gene Expression Analysis Using qRT-PCR

Samples for RNA extraction were prepared as previously described (Zhang et al., 2012) with some modifications. Briefly, conidia were first inoculated on plates containing Vogel’s liquid medium and grew for at least 24 h. The mycelial mat was then cut into fragments of about 2–4 mm^2^. About 10–12 such fragments were used to inoculate 150 ml flasks containing 100 ml liquid Vogel’s medium. The cultures were incubated at 28°C at 200 rpm. After growing for 13.5 h, antifungal agents were added and the cultures were further grown for 22 h. Since *erg11* is essential, the inactivation strain and its parental strain were grown in liquid Vogel’s medium containing 50 μM BCS during the first 13.5 h and then transferred to two new 150 ml flasks which were either unamended (control) or supplemented 200 μM CuSO_4_, respectively. The control flasks, but not the 200 μM CuSO_4_ flask also contained 50 μM BCS to support growth.

After 22 h of drug treatment, mycelial samples were harvested, flash frozen in liquid nitrogen, and ground into fine powder. Total RNA extraction was performed according to the standard TRIzol protocol (Invitrogen, Carlsbad, CA, United States). The cDNAs were synthesized from 3 μg total RNA using a cDNA synthesis kit (FastQuant RT Kit (with gDNase); TIANGEN, China) according to the manufacturer’s protocol.

Gene-specific primers were designed using the online tools PrimerQuest or Primer 6 and are listed in Supplementary Table 3. qRT-PCR was performed on a CFX96 multicolor real-time PCR detection system (Bio-Rad, Hercules, CA, United States) with SYBR green detection (KAPA SYBR^®^ FAST qPCR Kits; KAPA BIOSYSTEMS, Boston, MA, United States) according to the manufacturer’s instructions. Three independent experiments were carried out and each cDNA sample was analyzed at least in duplicate. The average threshold cycle (CT) values were used to calculate relative expression levels normalized to β-tubulin using the 2^-ΔΔ*CT*^ method (Livak and Schmittgen, 2001). The significance of the differences between each two samples were evaluated by an unpaired two-tailed Student’s *t*-test using GraphPad Prism 7.0 and a *p*-value of less than 0.05 was considered significant.

### ERG11 Detection by Western Blotting

For protein extraction, the collected mycelia were immediately frozen and then ground into fine powder for extraction. Proteins were extracted as previously reported (He et al., 2005) and quantified with the 2-D Quant Kit (GE, United States). Forty microgram protein of each sample was loaded for electrophoresis with SDS-PAGE and then transferred to a PVDF membrane (Merck Millipore, Germany). The tagged ERG11 protein level was detected using anti-myc antibodies (Abbkine, Redlands, CA, United States) using a standard protocol (Garceau et al., 1997).

### HPLC-MS Analysis of Sterol Contents and Ketoconazole

For analyzing intracellular sterols, mycelial samples were heat-dried at 80°C and then ground into fine powder for extraction. Extraction and analysis were performed as previously described (Sun et al., 2013) using ergosterol (J&K Scientific Ltd.) and KTC standards.

For analyzing extracellular sterols, the cultured medium was extracted by chloroform after lyophilization. The extractions were then analyzed by HPLC-MS as described (Sun et al., 2013).

The significance of the differences between each two samples were evaluated by an unpaired two-tailed Student’s *t*-test using GraphPad Prism 7.0 and a *p*-value of less than 0.05 was considered as significant.

## Results

### Generation and Characterization of a Strain with Inducible *erg11* Expression

In order to test whether transcriptional responses to azoles can be induced by disruption of ergosterol biosynthesis, we first used a genetic approach to impair the function of an essential enzyme in the pathway –sterol 14α-demethylase. To do so, we generated a strain, P*tcu-1*::*erg11*, in which the native *N. crassa erg11* promoter was replaced by the promoter of a copper responsive gene *tcu-1* (NCU00830, encoding the Ctr-domain containing copper transporter). When controlled by this promoter, expression of the target gene is repressed by high copper concentration while addition of the copper chelator BCS can release the suppression by copper (Ouyang et al., 2015).

The P*tcu-1*::*erg11* phenotype was characterized and compared to its parental *bd ku70^RIP^* strain. As shown in **Figure 2B**, on Vogel’s plates containing 50 μM BCS or 100 μM CuSO_4_, the growth of the parental strain was not affected, similar to that on normal Vogel’s plates. The growth of P*tcu-1*::*erg11* was severely inhibited on normal Vogel’s plate which containing 1 μM Cu^2+^, and only a small visible colony was formed (**Figure 2B**). The formed colonies of P*tcu-1*::*erg11* on the Vogel’s plates were also examined microscopically and clustered hyphae were observed (Supplementary Figure 1A). When 100 μM CuSO_4_ were added to Vogel’s plates, no visible growth of P*tcu-1*::*erg11* was detected and only short germlings were observed under microscope (**Figure 2B** and Supplementary Figure 1A). When copper chelator BCS were added to Vogel’s plates, the growth of P*tcu-1*::*erg11* was indistinguishable from that of the parental strain. Similar results were observed on Vogel’s slants amended with 100 μM BCS or 250 μM CuSO_4_ (**Figure 2B**).

When cultured in liquid medium containing 100 μM CuSO_4_, conidia of P*tcu-1*::*erg11* geminated normally (**Figure 2E**). However, after about 2 rounds of nuclear division, hyphal tips started to swell and grew no further. Nonetheless, nuclear division was not arrested (**Figure 2E**). As a result, multiple nuclei clustered together at the swollen hyphal tips. In the medium amended with the Cu^2+^ chelator BCS, hyphal growth of P*tcu-1*::*erg11* was not arrested and nuclei were distributed evenly along the germ tubes (**Figure 2E**), as observed in the parental strain (**Figure 2E**). This result indicates that *erg11* is required for hyphal elongation but not germination.

Since only the hyphal elongation was found to be affected in P*tcu-1*::*erg11* treated with Cu^2+^, the growth of the strains was examined by shifting the mycelial plugs from BCS to CuSO_4_ plates. As shown in Supplementary Figure 1B, after shifting to Vogel’s plates containing 100 μM CuSO_4_, the mycelium of P*tcu-1*::*erg11* strain grew for several hours and then ceased further colony growth, while the growth of parental strain was not affected. This result further verified that *erg11* is required for hyphal elongation, although the mycelium could grow for a while, possibly due to the presence of sufficient quantities of *erg11* mRNA or sterols within the mycelium to support growth.

**FIGURE 3:**
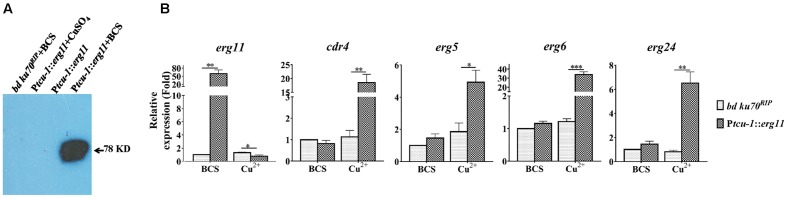
Transcriptional induction of *cdr4* and *erg* genes following *erg11* inactivation. **(A)** Detection of ERG11 (sterol 14α-demethylase) by Western Blotting in the P*tcu-1*::*erg11* strain grown in Vogel’s medium supplemented with Cu^2+^ or BCS using anti-myc antibodies. The parental strain (*bd Ku70^RIP^*) grown in Vogel’s medium supplemented with BCS and the P*tcu-1*::*erg11* strain grown in normal Vogel’s medium was used as controls. **(B)** After grown in medium containing BCS for 13.5 h, mycelium were then shifted to indicated conditions to grow for additional 22 h. The transcript levels of selected genes were examined in the *N. crassa bd Ku70^RIP^* strain and P*tcu-1*::*erg11* strain treated with 50 μM BCS and 100 μM CuSO_4_ for 22 h. Transcript levels of *cdr4* (NCU05591, encoding azole efflux pump CDR4), *erg5* (NCU05278, encoding C-22 sterol desaturase), *erg6* (NCU03006, encoding sterol C-24 methyl transferase), *erg11* (NCU02624, encoding sterol 14α-demethylase) and *erg24* (NCU08762, encoding C-14 sterol reductase) were measured by quantitative real-time polymerase chain reaction (qRT-PCR), and the expression was calculated by 2^-ΔΔ*Ct*^ method and normalized to β-tubulin. The results presented here are means of three biological replicates, and the significant levels between the two strains in each treatment were calculated by *t*-test and marked as ^∗^(*p* < 0.05), ^∗∗^(*p* < 0.01) or ^∗∗∗^(*p* < 0.001).

To verify that sterol 14α-demethylase ERG11 is affected in the P*tcu-1*::*erg11* strain treated with CuSO_4_, the sterol profile of this strain treated with CuSO_4_ was compared to that of the strain treated with BCS. As shown in **Figure 2D**, CuSO_4_ treatment resulted in the reduction of ergosterol to a level of approximately 56% of that treated with BCS along with the accumulation of lanosterol, eburicol and 14α-methyl-3,6-diol, similar to that found in *N. crassa* treated with azoles (Chen et al., 2016). The transcription and protein levels of *erg11* and ERG11 in P*tcu-1*::*erg11* in the presence of BCS and CuSO_4_, were also determined. In the presence of BCS, *erg11* expression was as high as 50 folds as that of the parental strain and the protein was easily detected (**Figure 3**). Mycelium pre-grown in the medium with BCS was transferred to the medium with CuSO_4_. The growth of the P*tcu-1*::*erg11* strain became slower than the parental strain. After 22 h at the time for RNA analysis, its growth was less than 50% that of the parental strain. Its *erg11* expression was twofold lower than the parental strain and the protein was undetectable (**Figure 3**).

All the phenotypic features described above indicate that *erg11* is an essential gene in *N. crassa*. To further verify that the observed effects were all due to the introduction of the inducible *erg11* cassette, P*tcu-1*::*erg11* was complemented by replacing the P*tcu-1* promoter with the native *erg11* promoter. Since P*tcu-1*::*erg11* could be inhibited by excess Cu^2+^, the transformants were screened on the medium containing 250 μM CuSO_4_ and verified by PCR. The complemented strains restored wild-type characteristics to the P*tcu-1*::*erg11* strain (**Figures 2B,C** and Supplementary Figure 1), verifying the essential roles of *erg11* for fungal growth.

### Inactivation of *erg11* Causes Transcriptional Responses Similar to Those Following Ketoconazole Treatment

To determine the relationship between the transcriptional response by the efflux-pump CDR4 encoding gene and ergosterol biosynthesis inhibition, we examined changes in *cdr4* expression when *erg11* was inactivated. In addition, we monitored the transcriptional changes of several *N. crassa* genes for ergosterol biosynthesis whose responses to azoles have been previously described (Sun et al., 2013). We compared the transcriptional responses between the P*tcu-1*::*erg11* strain and its parental counterpart when cultured in Vogel’s medium with BCS or CuSO_4_. When compared to the parental strain, BCS amendment did not affect most of the tested genes except for *erg11* in the P*tcu-1*::*erg11* strain. The presence of CuSO_4_ did not alter the abundance of any of the tested gene in the parental strain. However, down-regulation was observed in *erg11* expression measured in the P*tcu-1*::*erg11* strain cultured in the presence of the CuSO_4_ amendment, indicating that suppression of transcription of the gene regulated by the *tcu-1* promoter was functional (**Figure 3B**, left panel). However, a marked difference in transcript abundance of other tested genes was observed between the two strains grown in the presence of CuSO_4_. Transcript levels of *cdr4* as well as those of ergosterol biosynthesis genes were all significantly higher in the P*tcu-1*::*erg11* strain, in spite of the lack of KTC in the medium. While the growth of P*tcu-1*::*erg11* was inhibited after CuSO_4_ was added to the medium, the mRNA levels of *cdr4*, *erg5* (NCU05278, encoding sterol C-22 desaturase), *erg24* (NCU08762, encoding sterol C-14 reductase) and *erg6* (NCU03006, encoding sterol C-24 methyltransferase) were dramatically induced, by at least 28, 5, 6.5, and 33-fold, respectively, in a manner similar to the levels of induction imposed by the KTC treatment (**Figure 3B**). Hence, the presence of the drug was not a prerequisite for the transcriptional response observed.

### Ergosterol Depletion Is Not the Cause for Activating Stress Response

**FIGURE 4:**
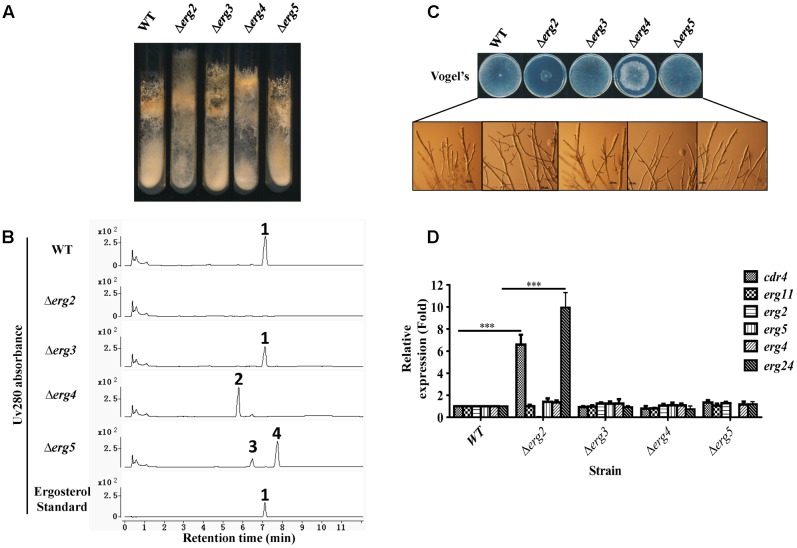
Phenotypic analysis and gene expression analysis of ergosterol deficient mutants. Conidia of *N. crassa* wild type, Δ*erg2* (NCU04156, encoding C-8 sterol isomerase), Δ*erg3* (NCU06207, encoding C-5 sterol desaturase), Δ*erg4* (NCU01333, encoding C-24(28) sterol reductase), and Δ*erg5* (NCU05278, encoding C-22 sterol desaturase) mutants were inoculated on Vogel’s slants **(A)** and Vogel’s plates **(C)** and grown at 28°C for 7 days **(A)** or 30 h **(C)**, respectively. **(B)** Major sterols in ergosterol deficient mutants. Mycelium grown in liquid Vogel’s medium for 36 h was used for sterol extraction. The extracted sterols were then subjected to HPLC-MS analysis. Ergosterol was identified by UV absorbance at 280 nm and referred to an ergosterol standard. Other sterols accumulated were further checked by MS and listed in Supplementary Table 4. The sterols marked with numbers are ergosterol for 1, ergosta-5,7,22,24(28)-tetraenol for 2, ergosta-5,7,24(28)-trienol for 3, ergosta-5,7-dienol for 4. **(D)** Transcript levels of *cdr4* (NCU05591, encoding azole efflux pump CDR4), *erg2*, *erg5*, *erg6* (NCU03006, encoding sterol C-24 methyl transferase), *erg11* (NCU02624, encoding sterol 14α-demethylase) and *erg24* (NCU08762, encoding C-14 sterol reductase) were measured by quantitative real-time polymerase chain reaction (qRT-PCR) in the selected mutants. The expression was calculated by 2^-ΔΔ*Ct*^ method and normalized to β-tubulin. The results presented here are means of three biological replicates, and the significant levels of *cdr4* and *erg24* between WT and Δ*erg2* were calculated by *t*-test and marked as ^∗^(*p* < 0.05), ^∗∗^(*p* < 0.01) or ^∗∗∗^(*p* < 0.001).

In order to determine whether the transcriptional responses are caused by ergosterol depletion in *N. crassa*, similar to that of other fungi, we took advantage of the available Δ*erg3* (NCU06207, encoding sterol C-5 desaturase), Δ*erg4* [NCU01333, encoding sterol C-24(28) reductase] and Δ*erg5* strains and produced another Δ*erg2* strain (**Table 1**). These genes encode enzymes involved in last few steps of ergosterol biosynthesis, and deletion or mutation in any of these genes results in ergosterol depletion (Grindle and Farrow, 1978; Sun et al., 2013; Weichert et al., 2016). All the mentioned gene deletion mutants are viable (**Figure 4A**), similarly to that described in *S. cerevisiae* (Lees et al., 1995). We first determined the extent of ergosterol deficiency in these mutants by sterol profiling. Similar as wild type, ergosterol was easily detected in Δ*erg3* strain, possibly due to the present of a second copy of *erg3* (Weichert et al., 2016). While in the Δ*erg2*, Δ*erg4* and Δ*erg5* strains, no ergosterol were detected (**Figure 4B**). Rather, ergosta-5,7,22,24(28)-tetraenol was accumulated in the Δ*erg4* strain and ergosta-5,7,24(28)-trienol and ergosta-5,7-dienol were accumulated in the Δ*erg5* strain when compared to wild type (**Figure 4B** and Supplementary Table 4). In the Δ*erg2* strain, ergosta-5,8,22-trienol and fecosterol were accumulated (Supplementary Table 4).

When cultured in slants, a marked reduction in conidial abundance was observed in the Δ*erg2* strain. Furthermore, aerial hyphae grew higher along the slant sides and in a more compact manner (**Figure 4A**). The Δ*erg2* strain grew extremely slow on Vogel’s plates, exhibiting a growth of about 25% of wild type (**Figure 4C**). A few more branches at the hyphal tips of this mutant were also observed (**Figure 4C**). Deletion of *erg4* also resulted in slow hyphal growth, albeit in a less severe manner than Δ*erg2*, as this strain grew approximately 80% of wild type, yet its colony morphology was more compact and fluffy in appearance (**Figure 4C**). Thus, deletions in two of the analyzed genes encoding enzymes catalyzing last steps in ergosterol biosynthesis resulted in growth defects, while deletion of the others (*erg3* and *erg5*) did not.

The expression of azole responsive genes including *cdr4*, *erg11*, *erg2*, *erg5*, *erg6* and *erg24* were then examined in these mutants by qRT-PCR. Interestingly, in a Δ*erg2* background, expression of *cdr4* and *erg24* were increased by 7 and 10-folds, respectively, while other genes tested were not affected (**Figure 4D**). This provides additional evidence that *cdr4* responds to ergosterol biosynthesis inhibition. However, no change in gene expression was evident in the mutants of *erg3*, *erg4* and *erg5*, which are downstream of *erg2* (**Figure 4D**). Thus, the stress responses to azoles by *cdr4* and ergosterol biosynthesis genes are not activated by ergosterol depletion.

### Differential Responses to Ergosterol Biosynthesis Inhibition in *N. crassa*

Above results indicate that inhibition at different steps in ergosterol biosynthesis, such as deletion of *erg2* or inactivation of *erg11*, could cause transcriptional responses by different genes. To further see transcriptional responses upon inhibition at more steps in ergosterol biosynthesis, we examined the transcriptional response of several genes to three different sterol biosynthesis inhibitors, KTC, AMOR and TERB. Different from azoles, TERB targets squalene epoxidase ERG1 (NCU08280), an enzyme upstream of sterol 14α-demethylase ERG11, while AMOR targets two enzymes downstream of ERG11 in the ergosterol biosynthetic pathway, sterol C8-C7 isomerase ERG2 and C-14 sterol reductase ERG24 (Lupetti et al., 2002) (**Figure 1**).

**FIGURE 5:**
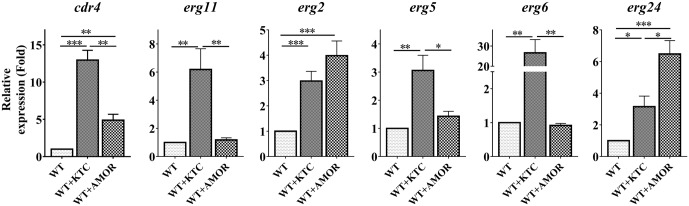
Differential transcriptional responses to different ergosterol biosynthesis inhibitors. After grown in liquid media for 13.5 h, the *N. crassa* wild-type strain were treated with 2 mg/L ketoconazole and 0.375 mg/L Amorolfine for 22 h, same amount dissolvent treatment was used as a control. Transcript levels of *cdr4* (NCU05591, encoding azole efflux pump CDR4), *erg2* (NCU04156, encoding C-8 sterol isomerase), *erg5* (NCU05278, encoding C-22 sterol desaturase), *erg6* (NCU03006, encoding sterol C-24 methyl transferase), *erg11* (NCU02624, encoding sterol 14α-demethylase) and *erg24* (NCU08762, encoding C-14 sterol reductase) were measured by quantitative real-time polymerase chain reaction (qRT-PCR), and the expression was calculated by the 2^-ΔΔ*Ct*^ method and normalized to β-tubulin. The results presented here are means of three biological replicates. The significant levels were calculated by *t*-test and marked as ^∗^(*p* < 0.05), ^∗∗^(*p* < 0.01) or ^∗∗∗^(*p* < 0.001).

As *cdr4* expression can be induced by inactivation of *erg11* or deletion of *erg2*, it was not surprising that AMOR or KTC treatments led to an almost 5- and 13-fold increase in *cdr4* expression, respectively (**Figure 5**), verifying that sterol inhibition does, in fact, induce *cdr4* transcription. TERB had no effect on *cdr4* expression (Supplementary Figure 2), suggesting that this pump may not be involved in expulsion of the compound from the cell, or, the inhibition of ergosterol biosynthesis by TERB cannot induce the expression of this pump.

We also analyzed the expression of several genes in ergosterol biosynthesis pathway, as affected by these antifungal drugs. As demonstrated above, transcription of these genes was induced by KTC (**Figure 5**). Similar to that observed in the Δ*erg2* strain, AMOR treatment only induced *erg24* by 6 folds but not other *erg* genes (**Figure 5**). AMOR treatment also resulted in 4-fold induction of *erg2*, the gene encoding one of the AMOR targets. Similar to that observed in the case of *cdr4*, no significant transcriptional responses of these tested *erg* genes to TERB were detected (Supplementary Figure 2). These results together indicate the responsive genes are different upon inhibition at different steps of the ergosterol biosynthesis pathway.

We then examined the sterol content changes in *N. crassa* treated with different ergosterol biosynthesis inhibitors. In the presence of all the inhibitors tested, ergosterol levels were reduced by at least two folds, excluding TERB which resulted in ergosterol depletion to only 80% of wild-type levels (Supplementary Figure 3 and Supplementary Table 4). Along with the depletion of ergosterol, KTC treatment resulted in accumulation of lanosterol, eburicol and 14α-methyl-3,6-diol, AMOR treatment resulted in accumulation of ignosterol and ergosta-5,8,22-trienol, while TERB treatment resulted in accumulation of squalene and an unknown compound (Supplementary Table 4). Thus, inhibition at different steps in ergosterol biosynthesis pathway resulted in accumulation of different sterol intermediates in addition to depletion of ergosterol.

### Stress Response Is Associated with Accumulation of Sterol Intermediates in *N. crassa*

Based on above results, it is possible that the responses by different genes could be associated with accumulation of different sterol intermediates. If this is the case, when the accumulated intermediates are reduced or other intermediates are additionally accumulated, the transcriptional responses caused may be reduced or changed.

To examine the possibility, we treated the Δ*erg2* strain with KTC (**Figure 1**). The sterol profiles were firstly compared between the two strains with or without KTC. When treated with KTC, the accumulation of lanosterol, eburicol and 14α-methyl-3,6-diol along with the depletion of ergosterol was observed in wild type (Supplementary Figure 4 and Supplementary Table 4). Deletion of *erg2* resulted in the accumulation of ergosta-5,8,22-trienol as its major sterol, while KTC treatment of this strain also lead to accumulation of lanosterol, eburicol and 14α-methyl-3,6-diol along with the depletion of ergosta-5,8, 22-trienol (Supplementary Figure 4 and Supplementary Table 4). Overall, KTC treatment in the Δ*erg2* strain could result in the depletion of sterol intermediates accumulated normally in the Δ*erg2* strain and the accumulation of sterol intermediates resulted from azole inhibition.

**FIGURE 6:**
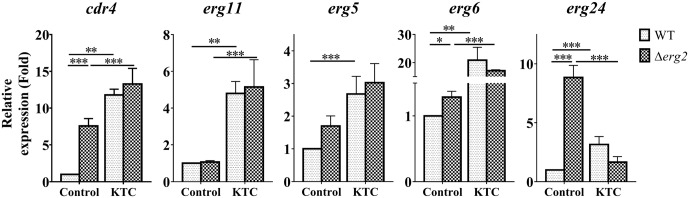
ERG11 inhibition overrides the effects of *erg2* deletion. After grown in liquid media for 13.5 h, the *N. crassa* wild-type and Δ*erg2* strains were treated with 2 mg/L ketoconazole for 22 h and same amount DMSO was used as a control. Transcript levels of *cdr4* (NCU05591, encoding azole efflux pump CDR4), *erg5* (NCU05278, encoding C-22 sterol desaturase), *erg6* (NCU03006, encoding sterol C-24 methyl transferase), *erg11* (NCU02624, encoding sterol 14α-demethylase) and *erg24* (NCU08762, encoding C-14 sterol reductase) were measured by quantitative real-time polymerase chain reaction (qRT-PCR), and the expression was calculated by 2^-ΔΔ*Ct*^ method and normalized to β-tubulin. The results presented here are means of three biological replicates. The significant levels were calculated by *t*-test and marked as ^∗^(*p* < 0.05), ^∗∗^(*p* < 0.01), or ^∗∗∗^(*p* < 0.001).

We then measured the expression of *cdr4* and *erg24* which respond to *erg2* deletion, *erg11* inactivation and corresponding antifungals. The expression of three additional genes, *erg11*, *erg5* and *erg6* which respond to only *erg11* inactivation or KTC inhibition, was also analyzed. As shown in **Figure 6**, transcription of *cdr4* was induced in a Δ*erg2* background. Yet when KTC was applied, the quantitative difference between the transcriptional responses of wild type and the mutant was minimal. Thus, it appears that the response of wild type to the drug is not dependent on a functional *erg2*. The expression of *erg24* in the Δ*erg2* mutant treated with KTC was reduced when compared to that in the Δ*erg2* mutant (nine folds) and similar to that observed in wild type treated with the drug. The expression of *erg5*, *erg6* and *erg11* in the Δ*erg2* mutant treated with KTC also showed a similar response to KTC induction on its own, which is very different from that in the non-treated Δ*erg2* strain. All the results indicate that the transcriptional responses by *cdr4* and genes in ergosterol biosynthesis pathway are associated with accumulation of sterol intermediates.

### Deletion of the *N. crassa* CDR4 Efflux Pump Leads to Intracellular, But Not Extracellular, Sterol Intermediate Accumulation Following KTC Treatment

**FIGURE 7:**
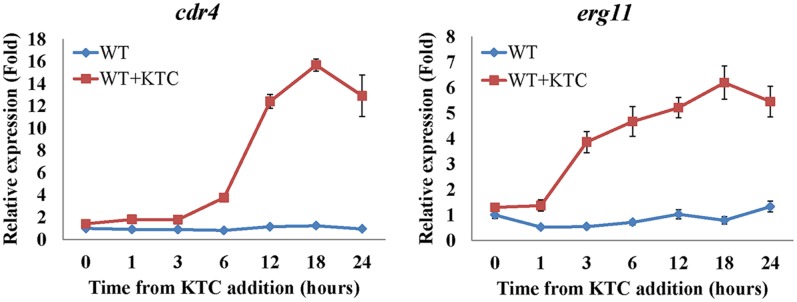
Transcriptional response of *cdr4* and *erg11* to 2 mg/L ketoconazole (KTC). After grown in liquid media for 13.5 h, KTC was added and grew for another 24 h. The gene expression of *cdr4* (NCU05591, encoding azole efflux pump CDR4) and *erg11* (NCU02624, encoding sterol 14α-demethylase) were monitored at various time points following KTC treatment. Transcript levels were measured by quantitative real-time polymerase chain reaction (qRT-PCR), and the expression was calculated with 2^-ΔΔ*Ct*^ method and normalized to β-tubulin.

During the course of this study we have found that even though *cdr4* expression can be induced by KTC and AMOR, when *erg11* expression is repressed or *erg2* is deleted, *cdr4* expression is significantly increased, even in the absence of the sterol biosynthesis inhibitor. Since the CDR4 homologs in *S. cerevisiae* and *C. albicans* have been shown capable of transporting steroids (Kolaczkowski et al., 1996; Krishnamurthy et al., 1998), it is possible that *N. crassa* CDR4 may also function as a transporter of sterol intermediates. As the first step toward analyzing the possibility, we examined whether the expression of *cdr4* is related to the accumulation of sterols. As inhibition or inactivation of *erg11* causes accumulation of lanosterol, eburicol and the toxic sterol 14α-methyl-3,6-diol (Watson et al., 1989; Chen et al., 2016) (Supplementary Figure 4 and Supplementary Table 4), we monitored *erg11* and *cdr4* expression along a 24-h period after amending the growth medium with KTC. As can be seen from the results of the time course study, the expression of *cdr4* was induced 3 h after KTC treatment while *erg11* expression was induced within 1 h in the presence of the drug (**Figure 7**). This suggests that the accumulation of the excess sterols occurs before the onset of pump activity.

**FIGURE 8:**
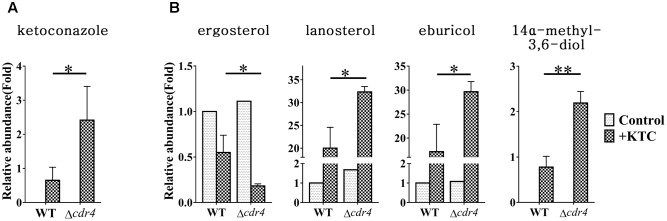
Sterol content between the *N. crassa* wild-type and Δ*cdr4* strain. After grown in liquid media for 13.5 h, the *N. crassa* wild-type and Δ*cdr4* strains were treated with 2 mg/L ketoconazole for 22 h and same amount DMSO was used as a control. KTC **(A)** and Sterols **(B)** were then extracted and measured by HPLC-MS. The abundance of sterols was calculated on the basis of the chromatogram peak area and normalized using an internal control and sample weight. The results presented here are means of three biological replicates. The significant levels were calculated by *t*-test and marked as ^∗^(*p* < 0.05), ^∗∗^(*p* < 0.01), or ^∗∗∗^(*p* < 0.001).

Do sterol intermediates accumulate in the Δ*cdr4* strain? To determine whether this is the case, we used HPLC-MS to analyze the sterol content in the *cdr4* deletion mutant with or without KTC treatment. Deletion of *cdr4* in *N. crassa* resulted in hypersensitive to azoles (Zhang et al., 2012). We firstly determined whether deletion of *cdr4* would result in the accumulation of KTC. The accumulation of KTC in the Δ*cdr4* strain was 2.4-fold of that in wild type (**Figure 8A**), confirming CDR4 is the transporter of azoles, functionally the same to its ortholog Pdr5p in *S. cerevisiae*. As shown in **Figure 8B**, under normal condition, deletion of *cdr4* had no obvious effect on the amount of intracellular sterols. In wild type, KTC treatment resulted in a reduction of total ergosterol content and the abnormal accumulation of sterols along the biosynthetic pathway (**Figure 8B**). In the Δ*cdr4* strain, a much more pronounced reduction (about threefolds) in total ergosterol content was evident, following KTC treatment (**Figure 8B**). This was accompanied by the increased accumulation of lanosterol, eburicol and the toxic sterol 14α-methyl-3,6-diol, which were 1.6-, 1.7- and 2.8-folds of that in wild type, respectively (**Figure 8B**).

To examine whether sterol intermediates can be exported via CDR4, the sterols in the cultured medium were also analyzed and compared between wild type and the Δ*cdr4* strain. Neither free ergosterol, nor lanosterol, eburicol or 14α-methyl-3,6-diol was detected in the media of any of the treatments. Moreover, other sterols, such as sterol acetate, were also not detected. Thus, the intracellular accumulation of sterols in Δ*cdr4* mutant is not due to reduced efflux of these sterols.

## Discussion

Transcriptional responses by the systems of efflux pumps and ergosterol homeostasis confer fungi the basal resistance to antifungal azoles (Cools et al., 2013; Paul and Moye-Rowley, 2014). Many studies suggest there is a linkage between the two systems in antifungal stress responses, but detailed evidence and investigation are required. Our results in this study showed that genetic disruption of *erg2* and *erg11*, two genes encoding the targets of ergosterol biosynthesis inhibitor amorolfine and azoles, respectively, activated the expression of azole efflux pump coding gene *cdr4*, indicating *cdr4* can make response to defects in ergosterol biosynthesis in absence of any ergosterol biosynthesis inhibitor. Azole-caused disruption in ergosterol biosynthesis should be an inductive factor for transcriptional response by *cdr4* to azole stress. Thus, this study reveals the linkage between the two systems in stress responses to azoles. It was reported that the expression of yeast azole efflux pumps could be activated directly by various drugs including azoles (Thakur et al., 2008). This study adds another mechanism to transcriptional activation of azole efflux pumps: indirect activation by azole-caused defects in ergosterol biosynthesis. Our results also showed that disruption at different steps in ergosterol biosynthesis had different effects and resulted in difference in sterol profiles. Interestingly, the transcriptional responses to disruption at different steps in ergosterol biosynthesis were also different, suggesting a correlation between the sterol profiles and transcriptional responses. Moreover, we found that abnormal ergosterol biosynthesis, causing accumulation of sterol intermediates but not ergosterol depletion, induced transcriptional responses by azole efflux pump coding gene *cdr4* and genes for ergosterol biosynthesis, providing a novel understanding to the mechanisms of stress responses to ergosterol biosynthesis inhibitors. Further studies by genome-wide transcriptional profiling might provide a comprehensive view on the responses and give more insights in how fungi cope with ergosterol biosynthesis inhibitors.

Our results clearly revealed that *cdr4* transcriptionally responds to defects in ergosterol biosynthesis. In *Kluyveromyces lactis*, a fungus more closely related to *S. cerevisiae* than to *N. crassa*, deletion of *ERG6* (encoding C-24 sterol methyl transferase) can also activate the expression of the CDR4 homolog (Konecna et al., 2016), providing another supporting evidence to the transcriptional activation induced by defects in ergosterol biosynthesis. Thus, the transcriptional induction of *cdr4* in *N. crassa* by compounds, such as xanthone derivatives, might be caused by their inhibition on ergosterol biosynthesis (Pinto et al., 2011; Pedro Gonçalves et al., 2015). Interestingly, DTT and avicel, which can induce ER stress, also activate the expression of *cdr4* in *N. crassa* (Coradetti et al., 2013; Fan et al., 2015). Since transcriptional induction of *cdr4* by DTT or avicel was shown to be independent on the canonical unfolded protein response (UPR) pathway that copes with ER stress (Fan et al., 2015), the transcriptional induction of *cdr4* might also be caused by inhibition on ergosterol biosynthesis. How efflux pumps and ergosterol biosynthesis link each other remains elusive. One possibility is that inhibition of ergosterol biosynthesis affects the function of the efflux pumps. In *C. albicans*, Cdr1p, the homolog of CDR4, prefers to be localized in lipid rafts which enrich in sterols and phospholipids (Pasrija et al., 2008). Thus, inhibition of ergosterol biosynthesis may disrupt the localization of efflux pumps and affect their function. In yeast, functional impairment by deletion of multiple efflux pumps, including CDR4 homolog Pdr5p, resulted in activation of the regulatory network of efflux pumps (Khakhina et al., 2015). Therefore, the activation of efflux pumps by ergosterol biosynthesis inhibitors might be a compensation effect of its functional loss caused by alteration in the sterol composition. Our results showed that accumulation of specific sterols but not ergosterol depletion was associated with the transcriptional response by *cdr4* (**Figure 1**). Although different sterols accumulated, those associated with the transcriptional induction of *cdr4* were sterols impaired in Δ^8^ to Δ^7^ isomerization (**Figure 1**). The role of this structure in the formation of lipid rafts is unknown, but defects in enzymes catalyzing this steps cause severe cell membrane associated impairment (Hannich et al., 2011). How this kind of sterols is associated with transcriptional responses by *cdr4* to ergosterol biosynthesis inhibitors needs further elucidation.

Genes in ergosterol biosynthesis are well known to respond to impairment in ergosterol biosynthesis. Genetic disruption of *ERG2*, *ERG5*, and *ERG6* in yeast can induce transcriptional responses by genes for ergosterol biosynthesis (Bammert and Fostel, 2000; Konecna et al., 2016). Ergosterol biosynthesis inhibitors that target Erg11, Erg24, Erg2 and Erg1 can also induce the responses (Bammert and Fostel, 2000; Henry et al., 2000). Responses to disruption at other steps in ergosterol biosynthesis were previously unknown. Surprisingly, we found that genetic disruption of *erg4* and *erg5* in *N. crassa* did not cause transcriptional response by any of the tested genes in ergosterol biosynthesis, although ergosterol formation was completely abolished. In addition, genes, responding to impairments in several specific steps in ergosterol biosynthesis, were different. The difference in responsive genes to different inhibitions on ergosterol biosynthesis was also found in *Trichophyton rubrum*, in which ketoconazole (KTC) upregulated most of the *erg* genes, such as genes encoding C-24 sterol methyl transferase Erg6, sterol 14α-demethylase Erg11 and C-14 sterol reductase Erg24, while TERB downregulated several *erg* genes and did not affect the expression of gene encoding sterol 14α-demethylase (Yu et al., 2007; Zhang et al., 2009). These results indicate the diversity in the responses to ergosterol biosynthesis inhibition among different fungi. A possible explanation for this is that, in yeast and *C. albicans*, ergosterol depletion alone can induce the responses. In *N. crassa*, depletion alone might be not sufficient to activate the responses. Induction of the responses might require more severe defects in the sterol profile, for example, accumulation of certain sterol intermediates. In humans, lanosterol accumulation promotes the degradation of HMG CoA reductase, an enzyme upstream of cholesterol biosynthesis (Song et al., 2005). Several studies have also proposed that abnormal sterol intermediates are responsible for the fungitoxicity of sterol biosynthesis inhibitors (Debieu et al., 1998). In *N. crassa*, accumulation of specific intermediates resulted in specific phenotypic outcomes (Weichert et al., 2016). However, it is not known which sterol intermediates may involve in the transcriptional responses. Based on our results, we proposed that 14-methyl sterols are associated with transcriptional responses to azoles (**Figure 1**). It is unclear whether different sterol intermediates can also result in different transcriptional responses in yeast, but exogenous addition of sterols with different structural features into the medium exerted different supporting effects on the growth of anaerobically cultured *S. cerevisiae* (Nes et al., 1978). Moreover, distinct defects are found in different *erg* mutants in yeast (Hannich et al., 2011). Thus, lack or aberrant accumulation of different ergosterol intermediates should cause the different functional defects in fungi and induce different transcriptional responses. However, how different intermediates activate transcriptional responses need further elucidation.

In summary, firstly, our data provide a novel explanation for how ergosterol biosynthesis inhibitors activate expression of efflux pumps: the responses of efflux pumps under drug stress are associated with the accumulation of sterol intermediate(s) resulting from defects in sterol 14α-demethylase ERG11 and sterol C-8 isomerase ERG2. However, why and how the efflux pumps are activated by sterol intermediates requires further elucidation. Secondly, our data also demonstrate that stress induction of ergosterol biosynthesis genes in *N. crassa* is also linked with sterol intermediates. This is markedly different from those observed in several other fungi, where genes for ergosterol biosynthesis are induced by ergosterol depletion, indicating different regulatory mechanisms among fungi. Such differences among fungi, especially between yeast and filamentous fungi, have been previously observed in several hierarchical and feedback systems, such as cell polarity, carbon catabolite repression and nitrogen regulation (Gorovits and Yarden, 2003; Harris, 2006; Wong et al., 2008; Ziv et al., 2008). Thus, in spite of similarities in the machinery involved, we demonstrate that the regulation and physiological outcomes in *N. crassa* are different from those observed in yeast and several other fungi and thus provide a novel explanation for the described aspect of antifungal stress response.

## Author Contributions

SL, CH, and OY designed the study and wrote the manuscript. CH and MZ performed the main experiments. WW performed the analysis of sterols. CH, XS, OY, and SL contributed to the data analysis and the data interpretation.

## Conflict of Interest Statement

The authors declare that the research was conducted in the absence of any commercial or financial relationships that could be construed as a potential conflict of interest.
