# Supplementary material for: Abnormal Ergosterol Biosynthesis Activates Transcriptional Responses to Antifungal Azoles
Source: Front Microbiol. 2018 Jan 17;9:9. doi: 10.3389/fmicb.2018.00009 (PMC5776110; doi:10.3389/fmicb.2018.00009)
Supplement: Supplementary file 2 [file Table_2.DOCX]

**Suppl Table 2. Primers used for plasmid construction in this study**

| Primer | Sequences  (5’→3’) | Use |
| --- | --- | --- |
| Ptcu-1F | TTCTAGAGGATCCTCTACGCCtGATGGGATAGAGAGAATGGCCGT | *erg11* promoter replacement by the *tcu-1* promoter and transformant analysis |
| Ptcu-1R | TCCCCCGGGCTGCAGGAATTCTTGGTTGGGGATGTGTGTGCG |  |
| HphR-F | GATAAGCTTGATATCGAATTCCCGTCGACAGAAGATGATATTGAAGGAG |  |
| HphR-R | GGCGTAGAGGATCCTCTAGAAAGAAG |  |
| exPerg-up-F | TGCGGTGCTGTTATTGCGAGG |  |
| exPerg-up-R | CTCCTTCAATATCATCTTCTGTCGACGGGCCGAGCCCTTTGTCACTCTACAC |  |
| exPerg-down-F | CGCACACACATCCCCAACCAAACAGGATCTGATATCATCGATTTAAAGCAATG |  |
| exPerg-down-R | GAGAGAAACCCATGTCGAGGTCG |  |
| hph-Ptcu-up-F | CCGTCGACAGAAGATGATATTGAAGGAG |  |
| hph-Ptcu-up-R | GTATTGACCGATTCCTTGCGGTCCGAA |  |
| hph-Ptcu-down-F | GATGTAGGAGGGCGTGGATATGTCCT |  |
| hph-Ptcu-down-R | TTGGTTGGGGATGTGTGTGCG |  |
| exPerg-vF | CATGATGGACCTGGCTGTGCTG |  |
| exPerg-vR | GCTGGTGGAGGACGAAGAATGC |  |
| TtrpC-F | CCGGGATCCACTTAACGTTACTGAA | Complementa-tion of the *erg11* inactivation strain |
| TtrpC-R | CATGCAAGAAAACAGGCGTAGAGGATCCTCTAGAAAGAAG |  |
| Perg11F | TCCTCTACGCCTGTTTTCTTGCATGTCTGAACCAAGC |  |
| Perg11R | TGATATCAGATCCTGTGGAGAAGGAGGCGGGGAGAAG |  |
| exPerg-down-F’ | CCTCCTTCTCCACAGGATCTGATATCATCGATTTAAAGCAATG |  |
| exPerg-down-R’ | Identical to exPerg-down-R |  |
| Perg2-up-F | CTCTGTTGCTCTCACGTCCTGC | *erg2* deletion |
| Perg2-up-R | CTCCTTCAATATCATCTTCTGTCGACGGGGTTTGTGATTGACGAGACGGC |  |
| Perg2-down-F | AGGAATAGAGTAGATGCCGACCGGACGCTAGACCGGAGTTTGGGTT |  |
| Perg2-down-R | GCCGAGTGCTGATCCTGAGTCT |  |
| hph-F | CCGTCGACAGAAGATGATATTGAAGGAG |  |
| hph-R | CCGGTCGGCATCTACTCTATTCCT |  |
