# Supplementary material for: Abnormal Ergosterol Biosynthesis Activates Transcriptional Responses to Antifungal Azoles
Source: Front Microbiol. 2018 Jan 17;9:9. doi: 10.3389/fmicb.2018.00009 (PMC5776110; doi:10.3389/fmicb.2018.00009)
Supplement: Supplementary file 3 [file Table_3.DOCX]

**Suppl Table 3. Gene specific primers used for qRT-PCR**

| Gene | Locus No. | Forward primer(5’→ 3’) | Reverse primer(5’→ 3’) | Ref |
| --- | --- | --- | --- | --- |
| β-tubulin | NCU04540 | CCCAAGAACATGATGGCTGCTTCT | TTGTTCTGAACGTTGCGCATCTGG | Chen, et al. 2016 |
| *erg2* | NCU04156 | TGAGCACCTTCACGATCTGTCCAA | TGATGTACATAGCACCCATGGCAC |  |
| *erg5* | NCU05278 | TTTCACCTTCCTCTTCGCTTCCCA | TCATCGACTCAAGCTGCTCCATGT |  |
| *erg11* | NCU02624 | AAATCGATTACGGCTACGGTCTCG | TATCGCTACCATCCACGTTCCTGA |  |
| *cdr4* | NCU05591 | GCTTTGGAAATGGATGGTGACGCT | AAATGCAGAGGGCGGTCTTAGAGT |  |
| *erg6* | NCU03006 | TCAGCTCAAGTTCGTCAAGGGTGA | TTCATAGACACCAAAGGTACCGCC | This study |
| *erg3* | NCU06207 | CGGTTCACCACTTCGCCTTCA | CATCCTCACCCTCCACCTCCTT |  |
| *erg24* | NCU08762 | TCATCGCCAAGCAATACC | CTCCGAAACTCAGCATGAA |  |
