# Supplementary material for: Abnormal Ergosterol Biosynthesis Activates Transcriptional Responses to Antifungal Azoles
Source: Front Microbiol. 2018 Jan 17;9:9. doi: 10.3389/fmicb.2018.00009 (PMC5776110; doi:10.3389/fmicb.2018.00009)
Supplement: Supplementary file 4 [file Table_4.DOCX]

**Suppl Table 4. Sterol profiles under different experimental conditions in this study.**

| Strain and Treatment | Major sterols | Sterol changes (fold^b^) | |
| --- | --- | --- | --- |
|  |  | Accumulated sterols | Depleted sterols |
| WT | ergosterol | NA | NA |
| ∆*erg2* | ergosta-5,8,22-trienol^a^ | NA | NA |
| ∆*erg3* | ergosterol | NA | NA |
| ∆*erg4* | ergosta-5,7,22,24(28)-tetraenol^a^ | NA | NA |
| ∆*erg5* | ergosta-5,7,24(28)-trienol; ergosta-5,7-dienol | NA | NA |
| P*tcu-1*::*erg11+*BCS | ergosterol | NA | NA |
| P*tcu-1*::*erg11+*Cu^2+^ | ergosterol | lanosterol (30.69±0.44), eburicol (172.86±1.09), 14α-methyl-3,6-diol (0.96±0.04^c^) | ergosterol (0.56±0.02) |
| WT+KTC | ergosterol | lanosterol (5.53±0.42), eburicol (17.42±1.01), 14α-methyl-3,6-diol (0.92±0.11^c^) | ergosterol (0.57±0.02) |
| ∆*erg2*+KTC | ergosta-5,8,22-trienol^a^ | lanosterol (9.49±1.38), eburicol (30.27±3.90), 14α-methyl-3,6-diol (1.04±0.01^c^) | ergosta-5,8,22-trienol (0.61±0.13) |
| WT+AMOR | ergosterol | Ignosterol (NA), ergosta-5,8,22-trienol (NA) | Ergosterol (0.40±0.04) |
| WT+TERB | ergosterol | squalene(NA) | Ergosterol (0.84±0.02) |

^a^ Fecosterol was also present, yet to a lesser extent.

^b^ The fold change is calculated as the relative amount to WT without fungicidal amendment, NA: Not Applicable;

^c^ The fold change of this sterol is calculated as the relative amount to WT treated with KTC or P*tcu-1*::*erg11* treated with Cu^2+^.
