# Supplementary material for: Abnormal Ergosterol Biosynthesis Activates Transcriptional Responses to Antifungal Azoles
Source: Front Microbiol. 2018 Jan 17;9:9. doi: 10.3389/fmicb.2018.00009 (PMC5776110; doi:10.3389/fmicb.2018.00009)
Supplement: Supplementary file 5 [file Image_1.PDF]

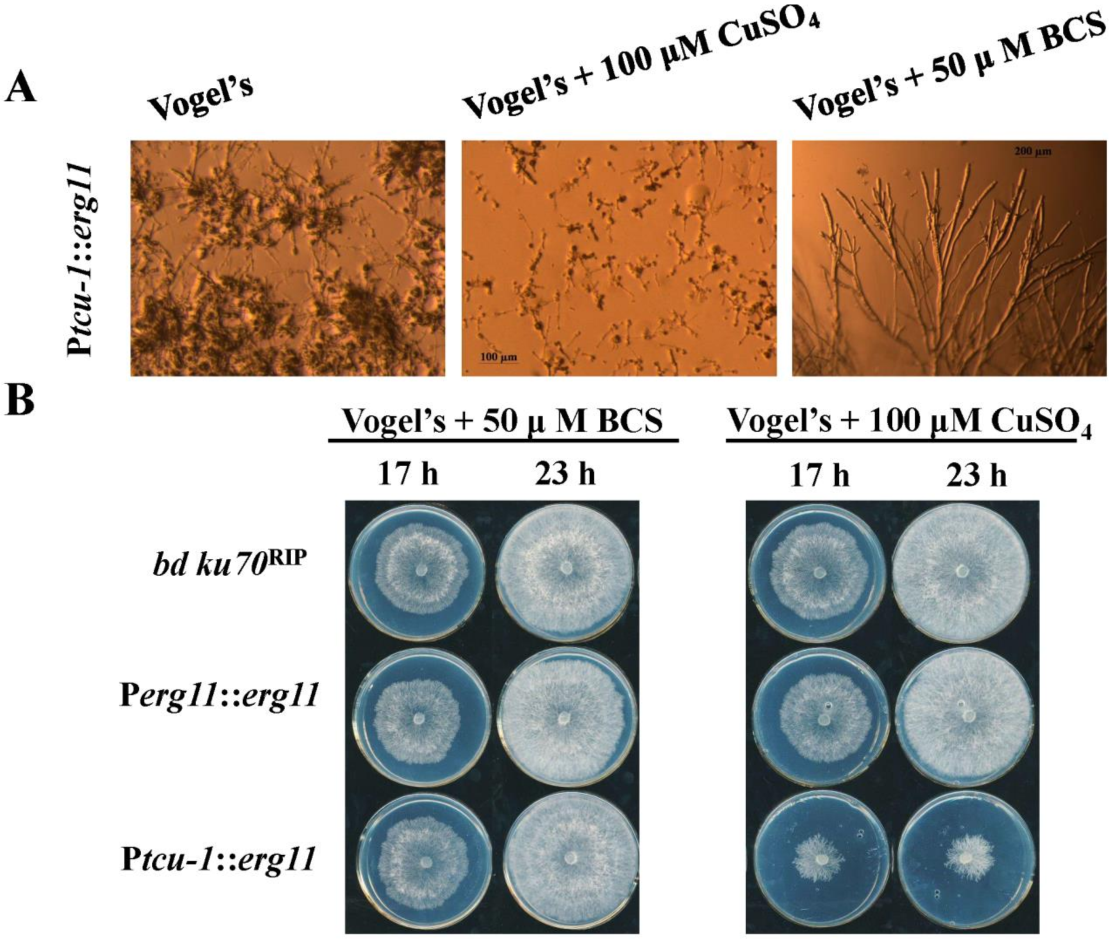


**Suppl Figure 1. *erg11* is required for hyphal growth in *N. crassa*. (**A) Colonies of *Ptcu-1*::*erg11* grown on plates with Vogel's, Vogel's+100 µM $CuSO_4$ and Vogel's+50 µM BCS media. (B) Growth of *bd ku70*$^{RIP}$, *Ptcu-1*::*erg11* and *Perg11*::*erg11* grown on Vogel's plates amended with BCS (left panel) or 100 µM $CuSO_4$. The respective strains were all first cultured on Vogel's plates amended with BCS. Hyphal growth was documented at the indicated time after plate inoculation with plugs of the various strains

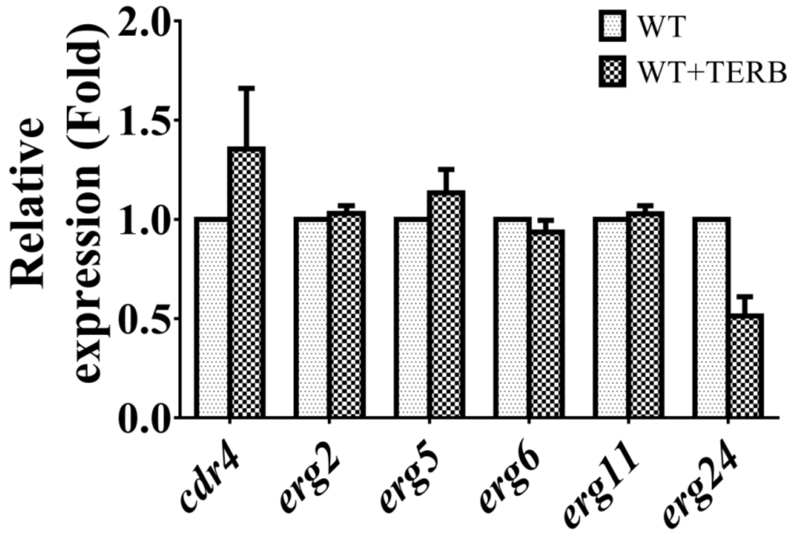


**Suppl Figure 2. Terbinafine has minor effects on the transcriptional induction of genes encoding efflux pumps and ergosterol biosynthesis enzymes.** After grown in liquid media for 13.5 h, the *N. crassa* wild type strain was treated with 3 mg/L terbinafine (TERB) for 22 hours (controls consisted of the same amount of the methanol solvent). Transcript levels of *cdr4* (NCU05591, encoding azole efflux pump CDR4), *erg2* (NCU04156, encoding C-8 sterol isomerase), *erg5* (NCU05278, encoding C-22 sterol desaturase), *erg6* (NCU03006, encoding sterol C-24 methyl transferase), *erg11* (NCU02624, encoding sterol 14α-demethylase) and *erg24* (NCU08762, encoding C-14 sterol reductase) were measured by quantitative real-time polymerase chain reaction (qRT-PCR), and the expression was calculated by the $2^{-\Delta\Delta Ct}$ method and normalized to β-tubulin. The results presented here are means of three biological replicates.

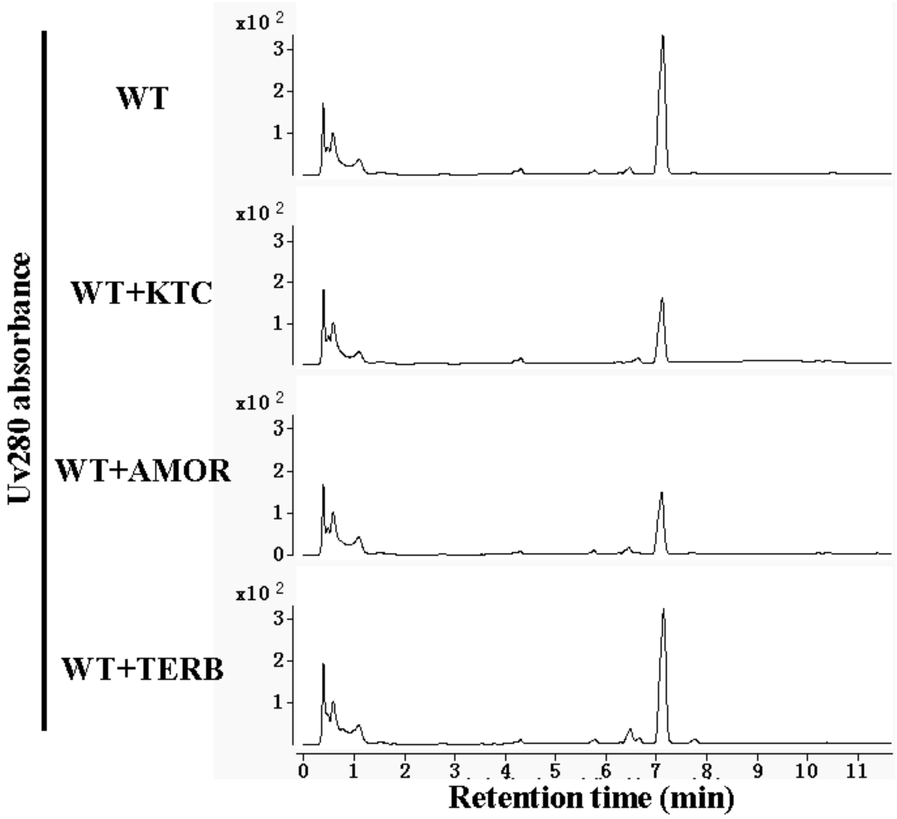


**Suppl Figure 3. Ergosterol depletion in *N. crassa* treated with different ergosterol biosynthesis inhibitors.** After grown in liquid Vogel's medium for 13.5 h, the medium was amended with 2 mg/L Ketoconazole, 0.375 mg/L Amorolfine or 3 mg/L Terbinafine and the strains grown for an additional 22 h, (see the legend of the previous figure). Sterols were then extracted and subjected to HPLC-MS analysis. Ergosterol was identified by UV absorbance at 280 nm and referred to an ergosterol standard. Other sterols were also analyzed by MS and are listed in Suppl Table 4.

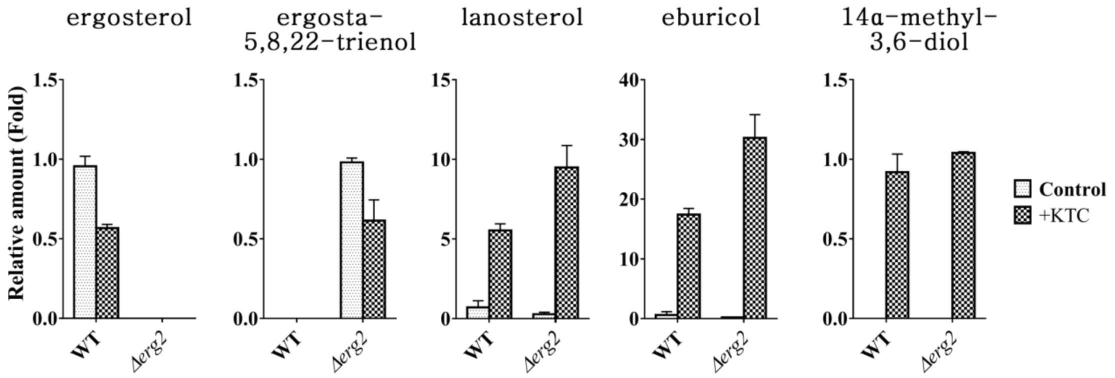


**Suppl Figure 4. Sterol content changes in Δ*erg-1* and wild type under azole stress.** After grown in liquid media for 13.5 h, the *N. crassa* wild type and Δ*erg2* strains were treated with 2 mg/L Ketoconazole for 22 hours (the same amount DMSO was used in control cultures). Sterols were then extracted and measured by HPLC-MS. The abundance of sterols was calculated on the basis of the chromatogram peak area and normalized using an internal control and sample weight. The results presented here are means of two biological replicates.
